# Dietary Therapy in Secondary Progressive Multiple Sclerosis: A Case Report

**DOI:** 10.7759/cureus.5341

**Published:** 2019-08-07

**Authors:** Janak Nathan, Dhanashri Khedekar Kale, Vidula D Naik, Forum Thakker, Sonal Bailur

**Affiliations:** 1 Neurology, Shushrusha Hospital, Mumbai, IND; 2 Clinical Nutrition & Dietetics, Dr. Nathan Sanjiv Clinic, Mumbai, IND

**Keywords:** secondary progressive multiple sclerosis, neurodegenerative disorder, ketogenic diet

## Abstract

A 60-year-old man presented with a history of an acute episode of mono-ocular involvement and several acute spinal cord episodes from 1988 to 1991. Multiple MRIs of the spinal cord and brain and cerebrospinal fluid analysis were consistent with a clinical diagnosis of multiple sclerosis (MS). Following this, there was a quiescent period of four to five years, after which he reported progressive weakness and spasticity of lower limbs with urgency and precipitancy of urine. He was put on a ketogenic diet (KD) as a monotherapy in 2016. Within one month of starting the KD, his balance and weakness improved, and there was good bladder control. He continued KD for 18 months, after which he followed it inconsistently and eventually stopped KD, going back to his original diet. His weakness increased gradually until he was wheelchair-bound, and his precipitancy greatly worsened. He was put back on KD and has improved again to the extent that his stamina has increased, he can walk with the help of a cane, and his continence is good. Dietary therapy has a large role in the management of secondary progressive multiple sclerosis (SPMS) and, as in this case, may be effective even as a single-mode therapy. This is probably the first reported case of improvement in SPMS using KD as a monotherapy.

## Introduction

The relapsing-remitting form of multiple sclerosis (RRMS) is an immunologically mediated demyelinating disease of the central nervous system (CNS) [1]. The progressive forms of multiple sclerosis (PMS), namely primary progressive multiple sclerosis (PPMS) and secondary progressive multiple sclerosis (SPMS), may have a different pathology [1]. Therapies used for RRMS are unsuccessful for PMS as neurodegeneration is seen without inflammation, and there is no disease-modifying medication at present, with many RRMS patients transitioning to SPMS within 15-20 years [2-3]. The ketogenic diet (KD) has beneficial effects in the treatment of neurodegenerative disorders, and could thus halt and reverse the progressive nature of PMS [2]. We report here a case of SPMS wherein the patient improved while on KD, worsened on stopping KD, and improved again upon restarting KD.

## Case presentation

A 60-year-old man had one episode of right mono-ocular blurring of vision in 1988 at 31 years of age. After two years, he had several acute episodes of right upper limb and right lower limb weakness. Inspite of not taking any treatment, good improvement was seen in several days. MRI scans showed fluid attenuated inversion recovery (FLAIR) hyper intense demyelinating lesions in various locations within the brainstem, the periventricular cerebral white matter, the infra-tentorial region of cerebellum, the subcortical cerebral white matter, and the spinal cord consistent with RRMS. The somato-sensory evoked potential studies, brainstem auditory evoked responses, and visual evoked responses were abnormal which were consistent with lesions in spinal cord, brain stem, and optic nerve respectively. The cerebrospinal fluid (CSF) analysis showed three oligoclonal bands (OCBs) but none in serum. The immunoglobin (IgG) index was also elevated (0.75) indicating inflammation of the CNS. All of the above parameters confirmed the diagnosis of multiple sclerosis (MS) as per the revised 2017 Macdonald criteria. There was no other possible etiology other than MS either clinically or on investigations. No further acute episodes were experienced for four to five years. Following this, there was progressive deterioration in his walking and balance with increase in spasticity, urgency of urine, and precipitancy. In 2015, he was put on the KD as a monotherapy. His baseline height was 165 cm and weight was 67.1 kg with an ideal body weight (IBW) of 65 kg. KD was started at a ketogenic ratio of 2.2:1 (ratio of fat to carbohydrate plus protein in grams) with 75% recommended dietary calorie allowance of 2600 Kcal and 25% calorie restriction (CR) to achieve IBW and later fine-tuned to maintain consistent urine ketones (UK) of 160 mg/dL (4+) and blood ketones on an average of 2.4 mmol/dL for 18 months. On KD, there was weight loss of 2 kg and his body weight was maintained on an average of 65 kg. Compliance and tolerability were good as ascertained by the patient’s diet diary and daily UK levels. He had no side-effects. He was assessed at regular intervals with the 25-foot walk, getting up from a chair, ascending and descending stairs, and expanded disability status scale (EDSS). Within a month, his balance and weakness improved and he maintained good bladder control. There was some deterioration observed in mid-2017 especially in walking and stairs test which could be attributed to slight irregularities in following KD. His physical evaluation is detailed in Table 1.

**Table 1:** Physical evaluation follow-up. Abbreviations: KD, ketogenic diet; EDSS, expanded disability status scale.

	Baseline July 2016	October 2016 (KD)	May 2017 (KD)	February 2018 (Stopped KD)	April 2018 (KD)	January 2019 (KD)
25-Foot walk	35 seconds	31 seconds	28 seconds	130 seconds	60 seconds	26 seconds
Walking (25 feet)	4 rounds	35 rounds	21 rounds	2 rounds	2 rounds	12 rounds
Getting up from chair (chair height- 22 inches)	30 times	45 times	54 times	7 times	7 times	63 times
Ascending and descending stairs	20 steps	100 steps	80 steps	2 steps	10 steps	20 steps
EDSS	6.0	6.0	6.0	7.5	6.0	6.0
Urine ketones	0 mg/dL	160 mg/dL	80 mg/dL	0 mg/dL	160 mg/dL	160 mg/dL
Blood ketones	0.2 mmol/dL	2.4 mmol/dL	1.4 mmol/dL	0.2 mmol/dL	1.3 mmol/dL	2.2 mmol/dL

After January 2018, he was inconsistent in following the KD and later stopped following it entirely. His strength deteriorated gradually until he could walk only a few steps with a walker and was mostly wheelchair-bound. He had to use diapers. His EDSS score was 6.0 which worsened to 7.5 after stopping KD (Table 1). On restarting KD, in March 2018, there was not much improvement for the first few months due to severe deterioration in physical abilities during the period he was off KD. Therefore the evaluation of April 2018 did not show much improvement in his rating scores. However his EDSS score (6.0) improved (Table 1). MRI scans done in 2017 and 2019 showed improvement on FLAIR with reduced intensity in lesions and no new lesions. However atrophy was seen on MRI in 2017 which worsened even further by 2019. The CSF analysis in 2017 showed two OCBs with none in serum. His IgG index remained elevated (0.7).

## Discussion

This patient had SPMS which showed definite improvement in EDSS scores, the 25-foot walk, and other parameters (Table 1). On stopping KD, all scores worsened; upon restarting KD, all scores improved.

Multiple sclerosis is an inflammatory disease with immune cells crossing a compromised blood-brain barrier [1]. However, in some cases of RRMS, neurodegeneration may be seen without inflammation, or there may be dissociation between the two processes as is also seen in Harding’s syndrome and Leber’s hereditary optic neuropathy [1]. This may be caused by an initial neurodegeneration causing an inflammatory reaction due to cellular degenerative material [1]. Also, in most PPMS and SPMS cases, immunomodulatory, immunosuppressive and autologous stem cell transplant therapies are not as effective, possibly because though they reduce inflammation, they have no effect on neurodegeneration [1-2]. Mitochondrial dysfunction plays a central role in PMS as is evidenced by abnormal mitochondria in damaged axons and cortical neurons and normal mitochondrial function in axons that escape demyelination [2]. Reduction in peroxisome proliferator-activated receptor gamma coactivator 1-alpha (PGC-1α) also occurs, a phenomenon common to other neurodegenerative diseases like Alzheimer’s disease [2]. Glucose hypometabolism is due to impaired mitochondrial metabolism and may play a significant role in disease progression in PMS [2, 4]. Thus, providing the brain with an alternative source of fuel like ketones may reduce the rate of neurodegeneration [2]. KD could be beneficial in the treatment of RRMS and PMS by various other mechanisms, too. KD suppresses inflammatory cytokines, increases CA1 hippocampal synaptic plasticity, and causes long-term potentiation which results in improved memory, learning, and motor ability in an RRMS rat model [5]. KD suppresses inflammatory cytokines by regulating the Janus kinase/signal transducer and activator of transcription pathway and exerting neuroprotective effect [6]. The anti-inflammatory effect of a KD might be explained through the inhibition of the NLRP3 inflammasome by beta-hydroxybutyrate (BHB) in a manner that is independent of starvation-induced mechanisms such as 5' adenosine monophosphate-activated protein kinase, autophagy, or glycolytic inhibition [2]. KD also reduces reactive oxygen species through uncoupling protein (UCP) activity, especially UCP2, UCP4, and UCP5 [2]. Subjects on a KD program had a modulation of microRNA’s targeting of specific genes thereby improving nutrient metabolism, signaling pathways, and neuroprotection [7]. Ketones are neuroprotective as they produce more energy in the form of adenosine triphosphate and reactive oxygen species production is reduced, thereby leading to less overall cellular stress for brain cells [2]. BHB has a direct, dose-dependent inhibitory activity on class I histone deacetylases (HDACs) including HDAC1, HDAC3, and HDAC4 [2]. KD raises glutathione levels in the hippocampus of rats through the nuclear factor erythroid 2-related factor pathway [2]. Mitochondrial biogenesis within the rat hippocampus and cerebellar vermis is increased by the KD [5]. Although the precise pathway for this is not known, it is thought to involve the PGC-1𝛼 family of transcriptional coactivators, which promote transcription factors including NRF-1, NRF-2, and ERR𝛼 [2]. There has been only one study done by Johns Hopkins School of Medicine on MS where they found that CR was feasible and led to safe weight loss in RRMS patients [8]. However, they did not analyze the efficacy of CR on treating RRMS symptoms [8]. Our patient improved on KD, his condition deteriorated when he stopped KD, and then improved again on restarting KD. There is a paucity of human studies on the use of KD in neurodegenerative disorders, and none in SPMS.

## Conclusions

This case raises the possibility that the mode of action of KD could represent a potential therapy for SPMS. However, large multi-center and randomized trials should be undertaken to better elucidate the complex relationship of KD vis-a-vis the etiopathology of SPMS.

## References

[1] Ontaneda D, Fox RJ, Chataway J (2015). Clinical trials in progressive multiple sclerosis: lessons learned and future perspectives. Lancet Neurol.

[2] Storoni M, Plant GT (2015). The therapeutic potential of the ketogenic diet in treating progressive multiple sclerosis. Mult Scler Int.

[3] Wingerchuk DM, Carter JL (2014). Multiple sclerosis: current and emerging disease-modifying therapies and treatment strategies. Mayo Clin Proc.

[4] Kindred JH, Tuulari JJ, Bucci M, Kalliokoski KK, Rudroff T (2015). Walking speed and brain glucose uptake are uncoupled in patients with multiple sclerosis. Front Hum Neurosci.

[5] Kim DY, Hao J, Liu R, Turner G, Shi FD, Rho JM (2012). Inflammation-mediated memory dysfunction and effects of a ketogenic diet in a murine model of multiple sclerosis. PLoS One.

[6] Hamid KM, Isiyaku A, Kalgo MU, Yahaya IS, Mirshafiey A (2017). JAK-STAT Lodges in multiple sclerosis: pathophysiology and therapeutic approach overview. OAlib.

[7] Cannataro R, Perri M, Gallelli L, Caroleo MC, De GS, Cione E (2019). Ketogenic diet acts on body remodeling and microRNAs expression profile. Microrna.

[8] Fitzgerald KC, Vizthum D, Henry-Barron B (2018). Effect of intermittent vs. daily calorie restriction on changes in weight and patient-reported outcomes in people with multiple sclerosis. Mult Scler Relat Disord.

